# Supplementary material for: Payments and freedoms: Effects of monetary and legal incentives on COVID-19 vaccination intentions in Germany
Source: PLoS One. 2022 May 24;17(5):e0268911. doi: 10.1371/journal.pone.0268911 (PMC9129024; doi:10.1371/journal.pone.0268911)
Supplement: S1 Table — (DOCX) [file pone.0268911.s001.docx]

**S1 Table. Determinants of getting vaccinated.**

| **Predictors** | **Getting vaccinated without monetary incentive** | |
| --- | --- | --- |
|  | *b* | *95% CI* |
| (Constant) | 0.11 | -0.105; 0.330 |
| Experimental manipulation: payment | **0.06** | 0.030; 0.106 |
| Experimental manipulation: legal incentive (Baseline: no legal incentive) | 0.02 | -0.034; 0.064 |
| Age | -0.00 | -0.003; 0.001 |
| Gender: female (Baseline: male) | -0.03 | -0.083; 0.018 |
| Education (Baseline: up to 9 years) |  |  |
| At least 10 years without university entrance qualification | 0.06 | -0.032; 0.146 |
| At least 10 years with university entrance qualification | 0.04 | -0.050; 0.132 |
| Household size (Baseline: 1 person) |  |  |
| 2 persons | -0.05 | -0.123; 0.014 |
| 3–4 persons | -0.03 | -0.101; 0.046 |
| More than 4 persons | -0.01 | -0.124; 0.107 |
| No answer | -0.10 | -0.965; 0.772 |
| Household income (Baseline: below 1.250 EUR) |  |  |
| 1.250–1.750 EUR | -0.04 | -0.144; 0.070 |
| 1.750–2.250 EUR | 0.05 | -0.051; 0.145 |
| 2.250–3.000 EUR | 0.09 | -0.005; 0.177 |
| 3.000–4.000 EUR | 0.05 | -0.047; 0.150 |
| 4.000–5000 EUR | **0.14** | 0.024; 0.253 |
| 5.000 EUR and more | 0.06 | -0.061; 0.171 |
| No answer | 0.02 | -0.103; 0.145 |
| Migration background (Baseline: yes) |  |  |
| No | 0.04 | -0.024; 0.113 |
| No answer | -0.02 | -0.307; 0.276 |
| Financial worries | -0.01 | -0.016; 0.007 |
| Confidence | **0.07** | 0.055; 0.086 |
| Complacency | **-0.05** | -0.073; -0.032 |
| Calculation | -0.01 | -0.018; 0.005 |
| Constraints | 0.00 | -0.016; 0.025 |
| Collective responsibility | **0.06** | 0.043; 0.080 |
| Payment x Experimental manipulation: legal incentive (Baseline: no legal incentive) | -0.01 | -0.014; 0.001 |
| Payment x Age | **-0.00** | -0.001; -0.000 |
| Payment x Gender: female (Baseline: male) | -0.00 | -0.011; 0.005 |
| Payment x Education (Baseline: up to 9 years) |  |  |
| At least 10 years without university entrance qualification | 0.01 | -0.003; 0.019 |
| At least 10 years with university entrance qualification | 0.01 | -0.004; 0.018 |
| Payment x Household size (Baseline: 1 person) |  |  |
| 2 persons | -0.01 | -0.018; 0.004 |
| 3–4 persons | -0.01 | -0.020; 0.004 |
| More than 4 persons | -0.00 | -0.019; 0.017 |
| No answer | -0.02 | -0.048, 0.010 |
| Payment x Household income (Baseline: below 1.250 EUR) |  |  |
| 1.250–1.750 EUR | 0.02 | -0.002; 0.034 |
| 1.750–2.250 EUR | **0.02** | 0.001; 0.034 |
| 2.250–3.000 EUR | 0.00 | -0.011; 0.018 |
| 3.000–4.000 EUR | 0.01 | -0.006; 0.027 |
| 4.000–5000 EUR | 0.00 | -0.014; 0.019 |
| 5.000 EUR and more | 0.01 | -0.006; 0.034 |
| No answer | 0.00 | -0.011; 0.019 |
| Payment x Migration background (Baseline: yes) |  |  |
| No | 0.00 | -0.008; 0.012 |
| No answer | -0.01 | -0.028; 0.015 |
| Payment x Financial worries | 0.00 | -0.001; 0.003 |
| Payment x Confidence | **-0.00** | -0.005; -0.001 |
| Payment x Complacency | -0.00 | -0.006; 0.002 |
| Payment x Calculation | 0.00 | -0.000; 0.003 |
| Payment x Constraints | 0.00 | -0.001; 0.005 |
| Payment x Collective responsibility | **-0.01** | -0.010; -0.002 |

*Note:* Results of linear regression analysis (*R*^2^ = .36) predicting willingness to get vaccinated (alternative to the analysis presented in S1 Table). Bold values denote significant predictors with *p* < .05.
