# Supplementary material for: Payments and freedoms: Effects of monetary and legal incentives on COVID-19 vaccination intentions in Germany
Source: PLoS One. 2022 May 24;17(5):e0268911. doi: 10.1371/journal.pone.0268911 (PMC9129024; doi:10.1371/journal.pone.0268911)
Supplement: S2 Table — (DOCX) [file pone.0268911.s002.docx]

**S2 Table. Determinants of getting vaccinated with and without monetary incentive.**

| **Predictors** | **Getting vaccinated without monetary incentive** | | **Getting vaccinated for monetary incentive only** | |
| --- | --- | --- | --- | --- |
|  | *OR* | *95% CI* | *OR* | *95% CI* |
| (Constant) | **0.06** | 0.006; 0.648 | 1.13 | 0.146; 8.715 |
| Experimental manipulation: legal incentive (Baseline: no legal incentive) | 1.01 | 0.577; 1.755 | 0.62 | 0.362; 1.059 |
| Age | 1.01 | 0.984; 1.027 | **0.98** | 0.959; 0.999 |
| Gender: female (Baseline: male) | 0.63 | 0.347; 1.129 | 0.79 | 0.442; 1.402 |
| Education (Baseline: up to 9 years) |  |  |  |  |
| At least 10 years without university entrance qualification | 2.37 | 0.977; 5.772 | 1.56 | 0.664; 3.664 |
| At least 10 years with university entrance qualification | 1.79 | 0.727; 4.408 | 1.78 | 0.746; 4.232 |
| Household size (Baseline: 1 person) |  |  |  |  |
| 2 persons | 0.83 | 0.384; 1.811 | 0.71 | 0.342; 1.479 |
| 3–4 persons | 0.73 | 0.297; 1.814 | 0.70 | 0.299; 1.638 |
| More than 4 persons | 1.15 | 0.243; 5.414 | 2.14 | 0.524; 8.766 |
| No answer | 0.25 | 0.008; 8.176 | **0.00** | 0.000; 0.000 |
| Household income (Baseline: below 1.250 EUR) |  |  |  |  |
| 1.250–1.750 EUR | 1.23 | 0.465; 3.244 | 1.62 | 0.657; 4.008 |
| 1.750–2.250 EUR | **5.08** | 1.652; 15.649 | **3.93** | 1.363; 11.351 |
| 2.250–3.000 EUR | 2.53 | 0.935; 6.818 | 1.40 | 0.533; 3.681 |
| 3.000–4.000 EUR | 2.65 | 0.884; 7.934 | 2.22 | 0.764; 6.442 |
| 4.000–5000 EUR | **4.36** | 1.308; 14.560 | 1.35 | 0.396; 4.611 |
| 5.000 EUR and more | 1.45 | 0.359; 5.848 | 1.28 | 0.330; 4.935 |
| No answer | 0.86 | 0.284; 2.599 | 0.64 | 0.199; 2.060 |
| Migration background (Baseline: yes) |  |  |  |  |
| No | 1.24 | 0.620; 2.486 | 1.02 | 0.524; 1.999 |
| No answer | 0.43 | 0.002; 73.788 | 1.84 | 0.095; 35.771 |
| Financial worries | 1.02 | 0.896; 1.156 | 1.02 | 0.913; 1.156 |
| Confidence | **2.32** | 1.942; 2.771 | **1.49** | 1.257; 1.769 |
| Complacency | **0.55** | 0.451; 0.683 | **0.81** | 0.688; 0.957 |
| Calculation | **0.80** | 0.677; 0.935 | 0.99 | 0.851; 1.159 |
| Constraints | 1.09 | 0.879; 1.358 | 1.07 | 0.894; 1.289 |
| Collective responsibility | **1.51** | 1.259; 1.804 | 0.99 | 0.851; 1.151 |

*Note:* Results of the multinomial logistic regression analysis (Cox & Snell’s *R*^2^ = .51, Nagelkerke’s *R*^2^ = .61). Both groups were compared to participants not willing to get vaccinated regardless of payment. Bold values denote significant predictors with *p* < .05.
