# Supplementary material for: Payments and freedoms: Effects of monetary and legal incentives on COVID-19 vaccination intentions in Germany
Source: PLoS One. 2022 May 24;17(5):e0268911. doi: 10.1371/journal.pone.0268911 (PMC9129024; doi:10.1371/journal.pone.0268911)
Supplement: S3 Table — (DOCX) [file pone.0268911.s003.docx]

**S3 Table. Determinants of minimum required monetary incentives.**

| **Predictors** | **Model 1** | | | | **Model 2** | | | |
| --- | --- | --- | --- | --- | --- | --- | --- | --- |
|  | β | *b* | *SE* | *95% CI* | β | *b* | *SE* | *95% CI* |
| (Constant) |  | 3,837.08 | 2,637.19 | –1331.737; 9005.870 |  | 3,543.28 | 2,176.94 | –1732.433; 6801.001 |
| Experimental manipulation: legal incentive (Baseline: no legal incentive) | **–0.35** | **–2,620.53** | 744.76 | –4080.235; –1160.826 | **–0.37** | **–2,550.45** | 561.49 | –3650.939; –1449.957 |
| Age | **0.24** | **60.08** | 26.81 | 7.531; 112.619 | **0.26** | **60.76** | 22.39 | 16.885; 104.637 |
| Gender: female (Baseline: male) | 0.11 | 810.57 | 762.21 | –683.344; 2304.474 | 0.08 | 539.54 | 627.05 | –689.443; 1768.532 |
| Education (Baseline: up to 9 years) |  |  |  |  |  |  |  |  |
| At least 10 years without university entrance qualification | –0.16 | –1,218.81 | 1,323.67 | –3813.153; 1375.526 | –0.14 | –964.34 | 1,094.85 | –3110.202; 1181.518 |
| At least 10 years with university entrance qualification | –0.14 | –1,076.25 | 1,279.96 | –3584.926; 1432.436 | –0.13 | –933.50 | 1,031.10 | –2954.427; 1087.422 |
| Household size (Baseline: 1 person) |  |  |  |  |  |  |  |  |
| 2 persons | **0.24** | **1,955.84** | 946.68 | 100.392; 3811.295 | **0.27** | **2,000.50** | 801.25 | 430.073; 3570.929 |
| 3–4 persons | 0.19 | 1,523.15 | 1,067.11 | –568.343; 3614.646 | 0.21 | 1,496.96 | 888.69 | –244.844; 3238.755 |
| More than 4 persons | 0.22 | 2,979.78 | 1,547.47 | –53.206; 6012.775 | 0.20 | 2,357.34 | 1,246.76 | –86.260; 4800.945 |
| Household income (Baseline: below 1.250 EUR) |  |  |  |  |  |  |  |  |
| 1.250–1.750 EUR | 0.10 | 942.32 | 1,160.15 | –1331.535; 3216.173 | 0.14 | 1,300.69 | 1,006.93 | –672.860; 3274.243 |
| 1.750–2.250 EUR | –0.04 | –382.25 | 1,240.43 | –2813.442; 2048.944 | –0.05 | –439.71 | 960.78 | –2322.806; 1443.379 |
| 2.250–3.000 EUR | –0.01 | –137.89 | 1,384.45 | –2851.370; 2575.588 | –0.01 | –125.27 | 1101.25 | –2283.681; 2033.137 |
| 3.000–4.000 EUR | –0.06 | –643.34 | 1,344.72 | –3278.950; 1992.274 | –0.05 | –506.79 | 1,102.02 | –2666.753; 1653.169 |
| 4.000–5000 EUR | –0.04 | –603.32 | 1,848.74 | –4226.786; 3020.151 | –0.01 | –105.87 | 1,388.10 | –2826.490; 2614.760 |
| 5.000 EUR and more | 0.01 | 99.37 | 1,839.27 | –3505.543; 3704.275 | –0.01 | –72.20 | 1,529.80 | –3070.559; 2926.161 |
| No answer | –0.05 | –824.17 | 1,782.38 | –4317.575; 2669.231 | –0.09 | –1,275 | 1,431.31 | –4080.464; 1530.149 |
| Migration background (Baseline: yes) |  |  |  |  |  |  |  |  |
| No | **–0.20** | **–1,770.20** | 832.61 | –3402.075; –138.316 | –0.16 | –1,279.15 | 668.15 | –2588.690; 30.396 |
| No answer |  |  |  |  | 0.01 | 471.31 | 3,431.96 | –6255.213; 7197.832 |
| Financial worries | –0.01 | –18.23 | 150.47 | –313.149; 276.693 | 0.03 | 47.48 | 125.62 | –198.728; 293.685 |
| Confidence | –0.08 | –175.31 | 205.76 | –578.589; 227.977 | –0.11 | –208.79 | 158.14 | –518.742; 101.168 |
| Complacency | –0.07 | –153.74 | 252.06 | –647.767; 340.292 | –0.11 | –203.61 | 197.67 | –591.046; 183.819 |
| Calculation | 0.02 | 48.47 | 204.64 | –352.626; 449.566 | 0.03 | 55.35 | 172.80 | –283.333; 394.037 |
| Constraints | 0.17 | 368.95 | 243.48 | –108.256; 846.155 | **0.20** | **395.50** | 198.72 | 6.007; 784.992 |
| Collective responsibility | –0.13 | –253.02 | 201.06 | –647.095; 141.048 | –0.10 | -174.58 | 164.17 | –496.337; 147.183 |

*Note:* Results of linear regression analyses (*R*^2^ = .357 and .324, adj. *R*^2^ = .203 and .193) predicting minimum required monetary incentives for those willing to vaccinate against payment only. In model 1, participants who switched between non-vaccination and (paid) vaccination more than once were excluded (*n* = 115). In model 2, their first switching point was interpreted as their minimum accepted payment (*n* = 143). Bold values denote significant predictors with *p* < .05.
