# Supplementary material for: Payments and freedoms: Effects of monetary and legal incentives on COVID-19 vaccination intentions in Germany
Source: PLoS One. 2022 May 24;17(5):e0268911. doi: 10.1371/journal.pone.0268911 (PMC9129024; doi:10.1371/journal.pone.0268911)
Supplement: Attachment — Submitted filename: Response to reviewers.docx [file pone.0268911.s004.docx]

**Response to Reviewers**

**Reviewer 1**

This study experimentally examines the influence of two factors – the presence/size of a financial incentive and whether vaccination would free individuals from testing requirements to engage in a range of social activities – on willingness to vaccinate in Germany. The experiment finds little evidence that the former matters; it finds some evidence that incentives can increase vaccination uptake, but only very large incentives.

The experimental design is clear, and the analysis is clearly and succinctly presented.

Thank you very much for your positive and helpful feedback. We revised and extended the manuscript. Please find our response to all your points below.

My main question/concern centers on the timing of the survey itself – in April of 2021. At the very least, the paper could do more to contextualize where the vaccination campaign stood in Germany at the time this survey went into the field. For example, I was initially surprised that >60% of unvaccinated respondents said they would receive a vaccine – until I went back and saw that the survey was fielded in April vs., say, November. The Discussion might engage a bit more the question of what lessons we can still draw about vaccination intention now when many more people have had the vaccine and the holdouts are perhaps even more steadfast in their opposition.

We added more context information to the paper. The reworked introduction now highlights that data was collected during a time when the majority of the German population were waiting for their first dose of a COVID-19 vaccine.

*“[…] we conducted a survey experiment investigating their single and combined potential effects on binary vaccination intentions in April 2021 in Germany. At that time, multiple vaccines were approved and recommended for the general population, but access was still restricted. As vulnerable groups and healthcare personnel were prioritized for vaccination, large parts of the population had to wait for their first shot, allowing us to investigate the impact of small and large payments as well as freedoms on vaccination intentions.”*

Although some time has passed since the experiment was conducted, the results are still timely and topical. First, we could show that large incentives can increase vaccination intentions and this may still be true. Furthermore, payments could play an important role in promoting booster uptake and speeding up vaccinations in future pandemics. The discussion has been updated accordingly:

*“Research conducted about a year after the presented study (and the large-scale rollout of vaccines to all Germans) suggests that the vast majority of unvaccinated individuals is unwilling to get vaccinated because they consider vaccination unsafe and identify with not being vaccinated [39]. Thus, it is unlikely that smaller payments are able to convince this rather extreme group. However, large incentives could still motivate some individuals to get the first shot. The present findings are important for future pandemics and recurring immunization: incentives could be used to promote booster uptake of already vaccinated individuals [40] and speed-up vaccinations in future pandemics.”*

I found the null results for the “freedom” treatment – vaccination can allow you to do a range of things without having to show proof of a negative test – particularly interesting. It also seems to dovetail with observational data from other countries. For example, I believe the vaccination campaign had slowed considerably in Italy when a negative test within 72 hours allowed you to obtain a Green Pass and engage in a wide range of activities… but many holdouts were converted and got vaccinated with the introduction of the Green Pass rafforzato and when this (which requires vaccination – not just a negative test) was made mandatory for a range of activities. The Discussion might engage such policy discussions a bit more directly.

Indeed, new data on the effects of vaccination certificates in Italy, France, and Germany supports what we previously only assumed in the discussion: legal incentives may only have an impact on vaccination uptake when testing is not treated equally to vaccination. The data thus is nicely in line with your observation. We updated the discussion accordingly:

*“Our results indicate that legal incentives do not increase the willingness to vaccinate. However, this may be due to the specific incentives offered in the experiment, where vaccination was predominantly framed as a replacement for testing. Offering stronger incentives, such as being allowed to eat out, travel for leisure, or attend a music festival, could indeed boost vaccination intentions as indicated by other experimental research [15] and a recent analysis showing that the introduction of vaccination certificates required to access specific venues such as restaurants and clubs in late 2021 increased vaccination rates in Italy, France, and Germany [34].”*

In sum, I think the study is well-done. My main suggestions are that the authors could do more to contextualize the findings and highlight for readers how these results from a relatively early stage of the vaccination campaign can still inform contemporary debates over boosters and the like.

Thanks again. We added more context information and updated the discussion as written above.

**Reviewer 2**

This paper studies the effects of incentives on willingness to take the COVID-19 vaccine in Germany. The study was conducted in Germany in April of 2021. The study varied two types of incentives: monetary and legal incentives. First, across participants, it compared willingness to get vaccinates when being vaccinated provided more freedoms to when it did not. Second, within-subjects, it elicited participants’ willingness to vaccinate themselves for 22 potential monetary payments. The study finds that willingness to get vaccinated is not affected by the freedoms that it could provide, but that incentives of over 3,000 Euro increase willingness to vaccinate.

Understanding the effectiveness of policies that aim to increase vaccination rates is important for policy makers worldwide. This study focuses on Germany and elicits willingness to vaccinate for a sample of 782 individuals, who are representative of the German population in age, gender, and region in which they live.

Thank you very much for the positive and helpful feedback. We revised and extended the manuscript. Please find our response to all your points below.

The authors have done extensive work on willingness to vaccinate (for COVID-19). What is the contribution that this paper makes? The authors’ previous work focused on smaller incentives, is the main contribution of the present work to focus on higher incentives? How does this work differ from the work published in PNAS focusing also on Germany by Kluver et al. (2021)? This work showed that small incentives increased intentions to vaccinate.

The introduction was revised to emphasize the purpose of the paper. Drawing from inconclusive evidence about monetary incentives (including the study by Klüver et al., 2021), we first intended to investigate the impact of larger payments:

*“While a hypothetical experiment with German participants at the end of 2020 suggested that payments of up to 200 EUR (about 240 USD) for getting vaccinated did not increase people’s intentions to get vaccinated [14], a different picture emerged in another German study conducted in March 2021, where hypothetical vouchers worth 25 EUR or 50 EUR (about 60 USD) increased the subjective probability of getting vaccinated by 1 or 2.2 percentage points [15]. Evidence from the US was inconclusive as well. While one study showed that vaccination intentions increased by 4.5 (13.6) percentage points after individuals were offered a hypothetical payment of 100 (500) USD [16], another article investigating the effects of real payments of 10 and 50 USD in May and July 2021 found insignificant overall effects [17]. Data from Sweden further showed that offering a 24 USD voucher increased vaccination uptake by as much as 4 percentage points [18]. While the effect of monetary incentives certainly depends on the pandemic context, the current uptake level, the local health system and cultural background, the heterogeneity of results may in part be explained by payment size. Offering larger incentives could be more effective than paying smaller sums for increasing vaccination intentions.”*

Second, we were interested in the potential effects of freedoms and their interaction with monetary incentives. The objectives and potential benefits of the research have been revised at the end of the introduction.

We agree that Kluever et al. (2021) conducted similar research (ours and their experiments were conducted at the same time). However, they used small incentives and measured effects differently. While we focus on a binary and more policy-relevant outcome (intending to get vs. not to get vaccinated), they assessed vaccination probabilities on a 10-point scale. Slight changes on this scale do not necessarily translate into higher vaccination uptake. Futhermore, we focused on legal incentives that could also be gained by getting tested whereas Klüver et al. focused on more extensive rights and freedoms. We addressed the differences in the revised introduction:

*“One study [15] showed that offering hypothetical freedoms for vaccination (such as being allowed to travel, visiting cinemas, restaurants, or concerts) increased the subjective probability of getting vaccinated by 2.5 percentage points in a German study conducted in March 2021. Given this rather small effect, the impact of legal incentives may be negligible, especially when the offered freedoms are less extensive, and when they can also be gained with a negative test (as was the case in Germany in most of 2020 and 2021).”*

Furthermore, we investigated the interplay between antecedents of vaccination (the 5C) and incentives (see section 3.2), an important feature for understanding when incentives may work or not. Such analyses are lacking in other studies (including the one by Klüver et al., 2021).

What motivated the design of such high incentives? Are there examples of companies or governments paying such large amounts of money for individuals to get vaccinated? Would there be political support for such measures?

As early as August 2020, Robert Litan called on governments to pay 1,000 USD for vaccination. Since then, others have supported the idea of higher incentives and some companies started paying their employees even higher sums. For instance, Coca Cola introduced the payment of 2,000 USD in the US and Böttcher AG, a large German supplier for office staples even offered 5,000 EUR (about 6,000 USD). The introduction was updated accordingly:

*“[…] Accordingly, some researchers advocated for giving people 1,000 USD [19] and companies like Coca Cola started paying employees 2,000 USD for getting vaccinated [20].”*

What effect sizes was the study powered to detect?

While most analyses (sections 3.1 and 3.2) were based on large samples and are thus well-powered, the regression analyses presented in section 3.3 only included 115 (model 1) or 143 (model 2) participants. Power analyses revealed that model 1 analyses were slightly underpowered, but model 2 analyses were well-powered. Accordingly, we addressed statistical power considerations when revising the results section:

*“It should be noted that model 1 was slightly underpowered to find small to medium effects. When considering an error probability of α = .05, a power of 1 – β = .90, and 9 predictors, at least 141 participants were required to find medium effects with f ^2^ = .15. While this limits the interpretability of insignificant findings in model 1, model 2 was well-powered and the reported findings should be considered robust.”*

What percentage of subjects switched multiple times overall in the price list? How are they treated in the main analyses (e.g., Figure 1 and Table 1)? Were there monetary values (or a treatment) for which switching occurred more often?

As indicated in the revised section 3.1, 84 (10.7%) of unvaccinated participants showed non-monotone vaccination intentions (i.e., opted for vaccination at some amount but switched to non-vaccination at a higher amount). Out of those, 44 participants switched multiple times. Furthermore, switching did not differ by treatment:

*“In total 44 participants (6%) changed intentions multiple times, i.e., switched between vaccination and non-vaccination more than once. The share of these multiple switchers did not differ between legal incentive conditions (p = .12; Fisher’s exact test).”*

Regarding the monetary values, there appear to be no specific values for which multiple switching occurred more often than others.

In section 3.1 (Figure 1), individual decisions were considered for each and every amount; multiple switchers were not recoded to monotone behavior nor removed (if not indicated otherwise in the text).

In section 3.2 (Table 1), the lowest amount for which participants were willing to get vaccinated was used for grouping. The description was updated accordingly:

*“To investigate factors associated with the decision to get vaccinated with or without monetary incentives, we divided the experimental sample into three groups: (1) participants not willing to get vaccinated regardless of whether payment was offered (n = 144), (2) participants willing to get vaccinated without payment (n = 495), and (3) participants willing to get vaccinated only when payment was offered (n = 143). Participants with non-monotone intentions and those who switched between vaccination and non-vaccination multiple times were included in the latter two groups, i.e., when intending to get vaccinated without payment but denying vaccination for money in (2), or when intending to get vaccinated for some amount (different from 0 EUR) but opting for non-vaccination at a higher amount in (3).”*

The data was collected in April of 2021, at that point what percentage of the population had access to vaccination? Was there already a concern that there would be significant hesitancy in Germany?

We revised the introduction to provide more information about the study context:

*“While researchers have discussed the ethics of both monetary and legal incentives for COVID-19 vaccine uptake [25–28] but empirical research on the effects of both interventions was inconclusive or unavailable, we conducted a survey experiment investigating their single and combined potential effects on binary vaccination intentions in April 2021 in Germany. At that time, multiple vaccines were approved and recommended for the general population, but access was still restricted. As vulnerable groups and healthcare personnel were prioritized for vaccination, large parts of the population had to wait for their first shot, allowing us to investigate the impact of small and large payments as well as freedoms on vaccination intentions.”*

Table 1 divides participants into 3 groups, why not analyze each decision and cluster standard errors at the individual level? A lot of data is not considered in the analyses with this approach. The coefficients of a multinomial logit are also more difficult to interpret. If the reason is that several subjects switched multiple times, the analyses could be conducted including and excluding these subjects, for example.

The objective of the analysis presented in Table 1 was to compare three groups of people; those willing to get vaccinated without payment, those willing to get vaccinated only when payment is offered (irrespective of the payment amount), and those not willing to get vaccinated at all. Running a single regression with legal and monetary incentives (and their interaction) predicting vaccination may help to quantify the effect of payments and provides easy to interpret coefficients, but it does not show the important differences between the groups. Our main goal here is to show how the groups differ with regard to key attitudes towards vaccination such as confidence, complacency, or collective responsibility—an important feature other articles do not provide. We therefore decided to keep the multinomial regression in section 3.2. However, we agree that coefficients are difficult to interpret, which is not the case for the regression you suggested. We believe that both approaches nicely complement each other, and have thus added the suggested analysis in the online supplement (see Table S1). It supports what has been reported in section 3.1 and thus was referenced there: while legal incentives play no important role in increasing vaccination intentions, payments have a positive impact:

*“The results were in line with a supplemental linear regression investigating the main and interaction effects of payments (with legal incentives, the 5C, and socio-demographic variables) on vaccination intentions, again showing that legal incentives play no important role while payments can have a positive impact (for details, see supplemental Table S1).”*

I fail to understand the extrapolated cost calculation (Section 3.4). What are the underlying assumptions? Would the incentives be paid to everyone? What are the dynamic issues that may arise (people may prefer to wait in anticipation of an incentive)?

We revised the results section to provide more details about the calculation:

*“As monetary incentives had a positive effect on vaccination intentions, we calculated implementation costs when offering the adult German population a one-time payment for initial vaccination (18 years and older, including those who have already been vaccinated; about 70 million people). Based on the aforementioned results, costs depended on the targeted vaccination uptake rate (Fig 2). While it would be possible to vaccinate about 70% of the adult population without payments, increasing vaccination uptake to 80%, as demanded by the World Health Organization at the time of our study (WHO, 2021), would require incentives worth at least 500 billion EUR.”*

We further reworked the discussion, explaining that if monetary incentives are introduced, they should also be paid retrospectively:

*“[…] in case policymakers decide to announce and introduce payments, they should also be paid retrospectively to already vaccinated participants to prevent vaccination-ready individuals from deferring immunization and to mitigate anger and backlash due to perceived unfairness.”*

The paper’s abstract and conclusion mention educational interventions. However, the paper does not study educational interventions, in the sense that it does not test whether increasing perceptions of safety and understanding the prosocial impact of the vaccine lead to lower hesitance. Perhaps it’s better to remove references to a policy recommendation that has not been tested?

We slightly rephrased the abstract and the discussion on alternative interventions for increasing vaccination uptake. While we indeed did not investigate the impact of educational campaigns, our results indicate that confidence in vaccine safety and feeling responsible for the collective are important antecedents of vaccination. As previous research has shown that educational efforts can foster these antecedents (see, for instance, Betsch, Böhm, Korn, & Holtmann, 2017), communication efforts are fundamental. We therefore decided to still promote them in the manuscript.
